# Creative Problem Solving and Social Cooperation of Effective Physical Therapy Practice: A Pioneer Study and Overview

**DOI:** 10.1100/tsw.2003.25

**Published:** 2003-04-28

**Authors:** Eli Carmeli

**Affiliations:** Sackler School of Medicine, Tel Aviv University, POBox 39040, IL-69978 Ramat Aviv, Israel

**Keywords:** physical therapy, action research, clinical education

## Abstract

Action research (AR) has an important role to play in educating physical therapists. Increasing efforts should be encouraged to instigate AR programs in physical therapy practice and clinical education. Such programs commonly require considerable effort and understanding by clinical instructors, and require adoption of new educational methods. AR programs can lead physical therapists and clinicians to be more questioning and reflective in evaluating practical questions regarding patient therapy and education. The purpose of this article is to educate the readers on the importance of AR and to provide a few relevant references on that topic. A specific study is described in this paper in which physical therapy clinical instructors participated in a structured workshop designed to demonstrate the values of AR and how such values can be incorporated in teaching their students. AR can lead to improved therapist-patient interaction and help solve specific practical problems arising during therapy sessions.

### INTRODUCTION

The main aim of physical therapists is to try and understand specific physical disabilities and movement dysfunctions in patients, to work with patients, and to improve their physical performance. Physiotherapists should continually question their personal attitudes, beliefs, perceptions, and values and consciously confront them[1]. Successful application of action research (AR) can provide some of the tools for improving physical therapy practice and can also lead to improved collaboration with colleagues in related medical professions. The concept of AR is straightforward: the researcher needs to study and continually assess personal professional attitudes and responses to specific situations. Moreover, the integral continuous feedback loop in

AR goes beyond investigation to action and reflection, reducing the traditional separation between research and practice. Because it is difficult to quantify or assess subjective values or attitudes, the validity and reliability of AR methodology is commonly regarded as suspect and highly questionable[2]. There is relatively little literature on these issues and it has been argued that judging reliability in such studies can only be carried out on a restricted basis[3,4]. It has also been claimed that validity in AR concerns not just methodology, but also personal issues[5], and therefore its methodological theory is sometime rejected. AR needs evaluation and analysis of decisions made during the study to facilitate judgment of validity[6]. Thus, there are often unsuccessful efforts to assess the relationship between theoretical and practical outcomes.

Hart and Bond (1995) [14] described four types of AR: experimental, organizational, professional, and empowering, which are all concerned in some way to improve standards of nursing care in hospitals.

This work investigated the "***empowering***" type, which mainly aims to raise consciousness. The purpose of this article is to educate the readers of the importance of AR by enhancing user-control and shifting balance towards more pluralism. The main value of AR is in understanding and changing the clinical behavior in general, and emphasizing its evidenced-based practice on a regular basis. This paper considers the role of clinical instructors in physical therapy and aims to describe a simple rationale for conducting “transparent research” in physical therapy practice. Such research is mostly qualitative and results in observations and conclusions that are not based on standard statistical procedures or other means of quantification. Application of AR approaches should encourage physical therapy clinicians, instructors, and students to develop improved responses to uncertainty and complex situations in practical therapy sessions with their patients.

### THE BACKGROUND TO ACTION RESEARCH

The main value in AR is in understanding and changing human phenomena in general (e.g., behavior and situations) and under specific context such as teaching physical therapy students, the course entitled “topographical anatomy” in particular. Few “action research” studies in education and sociology have been carried out by teachers and sociologists[7,8]. There is very little sociological research published regarding clinical instructors in the allied health professions in general or in physical therapy in particular[9,10]. Most ethnographical (i.e., social) research concerning medical practice is written by social scientists presenting themselves as outside observers and critics. The idea of AR is that the researchers, as the participants in the study (i.e., clinical instructors in physical therapy), observe themselves and consider their behavior and responses as insiders. Then they pursue action aimed at understanding and improving this behavior.

In the case described in this study, AR ("empowering" type) was implemented by a group of clinical physical therapy instructors (the “participants” or the "internal audiences").

The aim of this study was to increase the psychosocial awareness of a clinical instructor by implementing the empowering approach of AR; thus, to specifically examine the psychosocial aspects of AR on topographical anatomy, manual muscle testing, and goniometry (i.e., measurements of range of motion of joints). Interpreting psychosocial climate in a class environment means developing awareness of the inter-relationships, attitudes, and means of communication between students and their teacher[11]. The practice of psychomotor skills is common in physical therapy clinics. In order to try to determine the best method of teaching these skills, a structured study was conducted concluding with a 1-day workshop for clinical instructors as suggested by Gillespie[12]. In the workshop, the three common psychomotor skills (verbal, technical, and written) were blended into one mock-practice “case-study” in a small group format using a problem-based learning method[13]. However, it is important to distinguish between AR, which is a research methodology, and problem-based learning, which is essentially pedagogic.

## METHODOLOGY

### Background to the Method of “Empowering" Type of AR

This study was developed as a result of specific requests from several clinical and academic physical therapy instructors, who were unhappy with the end-products resulting from interaction with their students. They felt that their students, despite 20 weeks internship, were still not quite ready for the “real world” or for treating patients independently.

The clinical instructors grappled with how potential educational strategies could be used to facilitate student’s development during the 20-week internship.

### Participants

According to AR, there are two different audiences/participants — internal and external. The internal audience was clinical instructors (n = 18), who voluntarily participated in the study. All had at least 5 years experience as a clinical instructors working with physical therapy students in different internship fields. All of them recognized the information on which they based their change, which collaboration throughout the process should have ensured. For the external audience (academic mentors, n = 3), the context and the integrity of the AR were recognizable and reliable.

### Study Design

Our study followed the action research model of Hart and Bond[14] and had two main phases. The initial phase took place in a simulated practice setting followed by a group discussion and reading assignments. The physical therapy laboratory, like the therapy clinic, is a challenging workplace. Theories and methods derived from social and basic sciences, as well as from specific professional disciplines, need to be applied[15]. During the first phase we practiced teaching a topographical anatomy and goniometry class as described below. The second phase was carried out in a clinic. Structured observations of the routines of patients (i.e., assessment, screening, and patient’s education) adopted by the clinical instructors were followed by a discussion[16]. Three “participants” were selected confidentially as well as the mentor, who served as an outside (see below), but structured, observer. The mentor also observed other participants teaching. The rational of the methods (i.e., methodological approach) is presented in the Discussion section.

The clinical instructors were presented with a problem-based case study titled “topographical anatomy and range of motion (ROM) of the shoulder girdle following anterior dislocation”. This was followed by an open discussion to help the “participants” determine how to identify the major ROM problems, and discover gaps in their professional knowledge and skills, and suggest strategies to resolve those gaps. The mentor (i.e., external audience), rather than teach by formal lecture or demonstration, only guided and facilitated the problem-solving process, and acquisition of psychomotor skills.

## RESULTS

In the group activity, the “participants” tended to focus on the anatomy, physiology, and biomechanics of the “case” and were relatively insensitive to the feelings of their peers or the role-played “patient”. This led not only to conflict within the group dynamics, but also undermined the actual practice of physical therapy and restricted active participation to a limited number of “participants”. At the end of the case presentation and discussion, the “participants” were asked to provide a reflective report as encouraged by others[17,18] including a detailed description (verbal and written) of how social and group dynamics can contribute to the practice of physical therapy. The three outside observers and the lecturer then shared their observations with the group and commented on the overall group dynamics. The participants searched for material regarding AR in physical therapy as a homework searching and reading assignment. The clinical instructors expressed and negotiated their appreciation for the intellectual stimulation they received from the study day and also the background reading material. Shortly afterwards, a broad intellectual discussion was initiated and the “participants” started to consider and rationalize the merits of applying AR in physical therapy practice.

### DISCUSSION

Teaching AR should involve a mutual undertaking between the subjects being studied and the teacher. Those being studied (in our case the clinical instructors) were full partners in decisions to participate in the research, in setting project goals, in planning the research procedures, and in reviewing data as they were collected[19]. AR can provide clinical instructors with a reflective process that helps broaden and deepen understanding and personal actions[20]. AR can help to shape the future of the physical therapist, both as a compassionate practitioner and as a reflective human being. The foundation of AR may require integration of narratives at three levels: personal history (narrative story), social practice, and literary activity[21]. It is claimed that the analysis of narrative and the practice of literary activity can provide a theoretical mode that provides coherence within the broad discipline of medical informatics[22].

The use of a single-day workshop provides a basic approach to train clinical instructors how to teach real physical therapy students during their internship, and to help them improve their skills in managing human relationships in practical settings. We have found that such insight has the potential to minimize conflicts common to physical therapy practice. There is a common tendency for physical therapists to be competitive and dismissive of professional colleagues. The AR of our study showed that clinical instructors in a laboratory setting could be encouraged to learn integration of group-based methods of patient’s care with fundamental biological knowledge. Extensive discussions were encouraged regarding what conditions determine facts as accurate or objective in anatomy and ROM. The “participants” needed to combine an understanding of theoretical concepts (joint morphology, human kinetics, etc.), and social science concepts (communication, empathy, etc.) in ways that are acceptable and credible to physical therapists[23].

### Structured Observation

Three selected “participants” and the mentor observed the group dynamics of their discussion under structured instructions. Structured observation adopts a systematic approach using prepared schedules to record specific behavioral information[2]. The rationale for the structured observation, in particular during the second phase of the study, was that the clinical instructors claimed that almost everything that matters in the clinic involves basic science (a review by Shepherd and Jensen[24]). In practice however, the work of the professional physical therapy clinician also involves fundamental social science. Indeed, the work of physical therapists involves physical contact with their patients and family while maintaining emotional support and sensitivity to their patients’ needs, concerns, and expectations. However, the clinical instructors raised their concerns claiming that this aspect is partially or entirely ignored in the training of physical therapy students, and it also widely and unintentionally ignored by professionals in the field. The possible reasons of ignoring it are historically or tradition-related; social context (i.e., teacher-student hierarchy); or professional (curriculum-related). Issues such as family involvement in patient care or staff conflicts are viewed as annoying problems that interfere with physical therapy practice. There is minimal expression of such concerns as normal, necessary, or desirable in the acquisition of psychomotor skills in laboratory environments. The observations were less intended to address short-term problems arising in the daily operation of physical therapy clinics, but rather to consider a problem recognized by the "observers" as a serious and worthy of recognition for physical therapists in general and for the education of physical therapy students.

The second phase encouraged the clinical instructors to express and practice their own social and technical skills. Finally, the "students" were asked to evaluate their own performance and to assess it logically in order to identify, generate, and evaluate elements of logical argument. They need to be able to recognize and differentiate facts from illusions or assumptions, and to distinguish what is relevant. Self-examination, self-criticism, and verbal expression are the first stages in critical thinking and reflective practice based on these processes.

Inevitably there are some constraints to "empowering" type of AR. An "empowering" approach requires autonomous practitioners with the power to implement the resulting action. This is a particularly difficult approach in a multidisciplinary setting where participants are at different levels of autonomy.

#### Benefits of AR

The most important benefit from such an AR study is to provide better data and more detailed information about specific areas of physical therapy. The study demonstrated the potential for improving personal attitudes of physical therapists and how the biological knowledge of the therapist can find expression in a social context. It is often assumed in the literature that researchers adopt specific roles and maintain these throughout the period of self-investigation and self-criticism[25]. As "quasi-insiders" it is difficult, or even impossible for investigators to remain impartial or unbiased. However, AR provides better understanding of specific phenomena under study by using inside observers rather than outsiders. AR furthers understanding how different people, including the researchers, interpret various situations.

There is relatively little research on social aspects regarding physical therapy in clinical environments. AR raises issues of which social theories are meaningful in practice. Such a humanistic approach to knowledge is not always widely appreciated. There have been extensive debates in social science concerning what constitutes professionalism among health care providers, how medical competence should be defined, and how to assess variations in health care practices from community to community. These debates may shape important policy changes regarding health management. However, students as well as practitioners commonly ignore AR regarding improved performance and more meaningful humanistic approaches by the health providers. Our study provides an example of the value of applying AR and determination of professional attitudes based on creative problem solving and social cooperation as a foundation for more effective physical therapy practice.

### CONCLUSIONS AND SUMMARY

Professional training programs in physical therapy can benefit greatly from action and transparent research. AR can help bridge the cultural gaps between academic researchers and practicing physical therapists and help resolve differing interpretations of problems and their solution.

AR should become a regular way of life for clinical instructors to encourage an increased questioning of values, practices, and ideas for improving performance. The problem is not that academic scholars write in such a technically sophisticated way that practitioners (i.e., clinical instructors) are unable to understand, but rather that practitioners have been a neglected audience for academic research. For that reason, much of the published academic literature often seems irrelevant to practitioners. Physical therapists rarely explain their work in the language of sociological science. AR is transparent because the subjects under investigation are full partners in the process of collecting and analyzing data, unlike some academic research where data are secret until published and there is normally no accountability or explanation to the subjects under study. Practitioners can often provide better ethnographic (i.e., real world) insight and data than outside observers can generate through direct observation, assuming that the practitioners are sufficiently motivated to understand the theoretical issues that scholars are trying to investigate.

### BIOSKETCH

Eli Carmeli, P.T., Ph.D., is currently working in a full-time tenure tract position at the Physical Therapy Department, Sackler Faculty of Medicine, Tel Aviv University. He is investigating the aging process both on the cellular and clinical level.
